# Ultrafine Grinded and Silane Grafted Iron Ore Tailings as Reinforcing Filler of Styrene Butadiene Rubber

**DOI:** 10.3390/ma15051756

**Published:** 2022-02-25

**Authors:** Qian Liu, Qingguo Tang, Weiwei Zhao, Zhiyuan Su, Cong Liang, Xinhui Duan, Jinsheng Liang

**Affiliations:** 1 Institute of Power Source and Ecomaterials Science, Hebei University of Technology, Tianjin 300130, China; Liuqian_0327@163.com (Q.L.); zw18653723581@163.com (W.Z.); suzhiyuan970118@163.com (Z.S.); liang1097@126.com (C.L.); dxh1191984@aliyun.com (X.D.); liangjinsheng@hebut.edu.cn (J.L.); 2 Key Laboratory of Special Function Materials for Ecological Environment and Information, Hebei University of Technology, Ministry of Education, Tianjin 300130, China

**Keywords:** iron ore tailings, surface organic modification, ultrafine grinding, reinforcing filler, styrene butadiene rubber

## Abstract

In order to realize the high value-added resource utilization of solid waste and reduce the cost of rubber manufacturing, iron ore tailings (IOTs) were used as raw material to prepare a reinforcing filler of rubber through ultrafine grinding and surface organic modification techniques. We studied the effects of ball mill grinding conditions on the particle size and distribution of grinded iron ore tailings (G-IOTs). The effects of bis-(triethoxy-silyl-propyl)-tetrasulfide (Si69)-modified G-IOT (Si69-G-IOT) loading levels on the cure characteristics, static mechanical and dynamic mechanical properties of the styrene butadiene rubber (SBR) composites were also explored in this paper. The grinding and modification mechanism of IOTs and the combination of filler and SBR matrix were explored by grinding simulation of population balance model, X-ray diffraction analysis, Fourier transform infrared spectroscopy and scanning electron microscopy. The results showed that when grinding IOTs at 2000 r/min for 150 min, the particle size distribution of the resulting G-IOTs was the narrowest, with a D90 value of 4.42 μm. The tensile strength and elongation at break of SBR filled with 120 phr Si69-G-IOT were 14.97 MPa and 596.36%, respectively.

## 1. Introduction

Filler is the main raw material of the rubber industry. It can not only enhance the performance of rubber composites but reduce the amount of rubber raw materials required for production. Carbon black and silica constitute the traditional rubber-reinforcing filler, which consumes a large amount of fossil energy during production, causing environmental pollution and high cost [1]. With the increase in public environmental awareness, research on energy-saving and environmentally friendly fillers has become more serious. Natural silicate clay minerals including montmorillonite [2], attapulgite [3], kaolin [4,5,6], sepiolite [7,8,9] and other nano/submicron powders have been studied for application in rubber composite because of their rich natural reserves, low price and unique structure.

However, clay minerals generally have inorganic functional groups exhibiting relatively weak affinity and poor dispersion in polymers. Many researchers have studied the effects of reinforcement with silane-grafted clay minerals on the mechanical properties of synthetic rubber composites. Tang et al. [10] used mercapto silane coupling agent to modified palygorskite nanofibers as a reinforcement to fill cis-polybuta-diene rubber. The tensile strength and tear strength of the rubber composite reached 12.97 MPa and 65.93 kN·m^−1^ at a filling amount of 70 phr. Tang et al. [11] also used 100 phr of titanate coupling agent-modified attapulgite to reinforce ethylene-propylene-diene monomer. The tensile strength of the rubber composite reached a maximum value of 18.32 MPa, and the optimal content of titanate was in the range of 2–3%. The mechanical properties of synthetic rubber have been significantly improved by filling large amount of clay minerals. It is possible to use clay minerals instead of traditional fillers in industrial production. However, clay minerals also have some problems, such as complex purification technology and shortage of mineral resources. The development of green, natural and low-cost rubber reinforcing filler is still worthy of further exploration and research.

Iron ore tailings (IOTs) are a typical industrial solid waste made up of oxides or clay silicate minerals such as quartz, feldspar, pyroxene, hornblende, etc. [12]. By 2018, the stockpile of tailings reached 5 billion tons in China [13]. This stockpile not only occupies cultivated land but causes serious pollution to air and water [14,15]. The large-scale resource utilization of IOTs mainly includes rebeneficiation of valuable elements and mine backfilling. With the construction of green and waste-free mines, especially the implementation of the limited mining of river sand policy, the utilization of iron ore tailings as a building material has been effectively promoted. Coarse IOT sand was used as sand and stone material for construction and produced permeable bricks, sidewalk bricks [16,17,18,19,20,21], cement raw materials [22,23,24], concrete [25,26], foamed ceramics and glass ceramics [27,28,29,30]. However, the high value-added industrial utilization of microfine iron tailings sludge has become an urgent problem that need to be solved. In recent years, microfine iron tailings have been researched as a filling for thermoplastic because of their fine particle size, high stiffness and similar composition to that of clay minerals. Adedayo et al. [31] prepared iron ore tailing–polypropylene composites. It was found that increasing the filling volume of IOT increased the impact strength of the composites. Onitiri et al. [32] used IOTs with different particle sizes to fill epoxy resin and found that the thermal conductivity of the composite was improved. When the IOT particle size was 300 µm and the filling volume fraction was 16.5%, the impact resistance was the strongest. Piffer et al. [33] filled maleic anhydride-grafted polypropylene with IOT and found that the crystallinity of the prepared composite was improved. These studies proved that IOT was a promising raw material for polymer fillers, in which prepared polymer composites may find applications in insulation and shock absorption of electrical equipment. It is worth noting that iron tailings contain a certain amount of iron oxide, which has the prospect of preparing magnetic polymer composites such as magnetorheological elastomers [34,35,36].

Styrene butadiene rubber (SBR) was obtained by free radical-initiated emulsion polymerization or anionic solution polymerization using butadiene and styrene as monomers. It is a versatile synthetic rubber variety with high capacity and consumption in the world. Like most nonpolar rubber, styrene butadiene rubber is unable to crystallize because of its irregular molecular structure. The existence of a benzene ring and vinyl give it poor flexibility and large internal friction in the rubber molecular chain, which result in its low strength as raw rubber [37]. Styrene butadiene rubber could not be applied without reinforcement with a large number of fillers.

In order to adapt to the carbon peak, carbon neutralization and sustainable green development policy, promoting the resource utilization of fine tailings is helpful. We used microfine IOT particles with composition and structure is similar to those of clay minerals as raw materials to prepare reinforcing filler through ultrafine grinding and surface organic modification techniques. We explored the effect of grinding conditions on the particle size distribution of IOTs. Further investigation focused on the effects of different filler loadings on SBR reinforcement.

## 2. Experiments

### 2.1. Chemicals and Materials

The microfine particle iron ore tailings (D_50_ = 28.91 μm, D_90_ = 88.62 μm, specific surface area 13.82 m^2^/g) used in the experiment were supplied by Luanping Jianlong Mining Co., Ltd., Chengde, China. Table 1 shown the main chemical components of the IOTs.

Styrene butadiene rubber (1502E-SBR) with styrene content of 23.5 wt%, Mooney viscosity (ML_1+4_, 100 °C) of 52 M and specific gravity of 0.933 g/cm^3^ was produced by Yousuo Chemical Technology Co., Ltd., Linyi, China. N-isopropyl-N’-phenyl-1,4-phenylenediamine (4010NA), zinc oxide, stearic acid, tetramethylthiuram-disulfide (TMTD), 2-mercaptobenzothiazole (M), sulfur and Vivatec 700-type environment-friendly aromatic hydrocarbon oil were supplied by Jingdong Rubber Co., Ltd., Baoding, China. The coupling agent bis-(triethoxy-silyl-propyl)-tetrasulfide (Si69) was produced by Nanjing Chuangshi Chemical Auxiliary Co., Ltd., Nanjing, China.

### 2.2. Characterizations

The chemical components of the IOTs were obtained using a ZSX Primus 2 X-ray fluorescence spectrometer (Rigaku, Tokyo, Japan).

The X-ray diffraction (XRD) measurements of IOT and G-IOT samples were obtained using a Smart Lab X-ray diffractometer (Hitachi, Tokyo, Japan) with Cu Kα (λ = 0.5418 Å) radiation operated at an accelerating voltage of 40 kV, an electric current of 150 mA, a scanning speed of 12 °/min and a 2θ angle ranging from 3 to 70°.

Fourier transform infrared (FTIR) spectroscopy of IOT, G-IOT and Si69-G-IOT samples was performed using a BUKER-80V Fourier transform infrared spectrometer (Brooke, Karlsruhe, Germany) in the range of 4000–400 cm^−1^ in KBr pellet form.

The particle size distributions of IOT and G-IOT samples were measured using a Mastersizer 2000 laser particle size distribution analyzer (Malvern Panalytical, Shanghai, China) with a particle refractive index of 1.52 and an absorption rate of 0.1.

The specific surface areas of IOT and G-IOT samples were obtained by nitrogen adsorption–desorption analysis using an Autosorb-iQ2-TPX physical adsorption analyzer (Quantachrome, Boynton Beach, FL, USA) at −196 °C according to the BET mathematical model.

The morphologies of the tensile fracture surfaces of SBR composites were obtained using an S4800 scanning electron microscope (Hitachi, Tokyo, Japan) at an accelerator voltage of 15.0 KV.

The cure characteristics of SBR compounds were measured using an MDR-2000E moving die rheometer (Liyuan Electronic Chemical Equipment, Wuxi, China) at 140 °C for 15 min. Scorch time (t_02_), optimum cure time (t_90_), maximum torque (M_H_) and minimum torque (M_L_) were measured. The cure rate index (CRI) was expressed as Equation (1), which indicates the rate of the vulcanization reaction.

(1) $$\mathrm{CRI}=\frac{100}{t_{90}-t_{02}}$$

The static mechanical properties of SBR composites were measured by a CMT 6104 Electromechanical Universal Testing Machine (SANS, Shenzhen, China) at room temperature with a crosshead speed of 500 mm/min a measuring range of the force sensor of 1000 kN. The tensile strength was measured according to standard GB/T 528-2009/ISO 37: 2005 with dumbbell (Type 1)-shaped specimens. The tear strength was measured according to standard GB/T 529-2008/ISO 34-1: 2004 with unnicked 90° angle-shaped specimens. The mechanical property data were the mean values of five repeated measurements.

The shore A hardness of SBR composites was calculated by an LK-A hardness tester (Jiangdu Daochun Experimental Machinery Factory, Yangzhou, China) according to GB/T 23651-2009/ISO 18517: 2005. The average of five readings is reported for each sample.

The dynamic mechanical analysis (DMA) of SBR composites was conducted using a Tritec 2000 dynamic mechanical analyzer (Triton technology, Nottinghamshire, UK) in tension mode at a prestrain of 0.5% and a dynamic strain of 1%, a test temperature from −80 °C to 80 °C, a heat rate of 3 °C/min and a frequency of 10 Hz.

### 2.3. Preparation of G-IOT/Si69-G-IOT Powders

#### 2.3.1. Wet Ultrafine Grinding of IOTs

Aqueous suspensions with 20 wt% solid content of IOT particles were ground in a mill pot. Zirconia balls with sizes of 2 and 5 mm were used as the grinding media. The total amount of the zirconia beads’ (2 mm/5 mm = 1:1) filling mass was three times that of the IOTs. The IOTs were grinded by a TJX-450 planetary ball mill (Dongfang Tianjing Technology Development Co., Ltd., Tianjin, China) with grinding speeds of 1000, 1500, 2000 and 2500 r/min for 30, 60, 90, 120 and 150 min. After suction filtration and drying at 105 °C for 10 h, the G-IOTs were dispersed through a 100-mesh sieve.

#### 2.3.2. Surface Modification of G-IOTs

As above, IOTs were ground with a ball mill grinding speed of 2000 r/min for 150 min to obtain aqueous suspensions of G-IOTs. The silane coupling agent Si69 with 5% of the IOT mass fraction was diluted with 10 mL of absolute ethanol and then added to the ball mill pot. The ball mill grinding speed was set to 1000 r/min for 5 h. After suction filtration and drying at 105 °C for 10 h, the Si69-G-IOTs were dispersed through a 100-mesh sieve.

### 2.4. Preparation of SBR Composites

**Figure 1:**
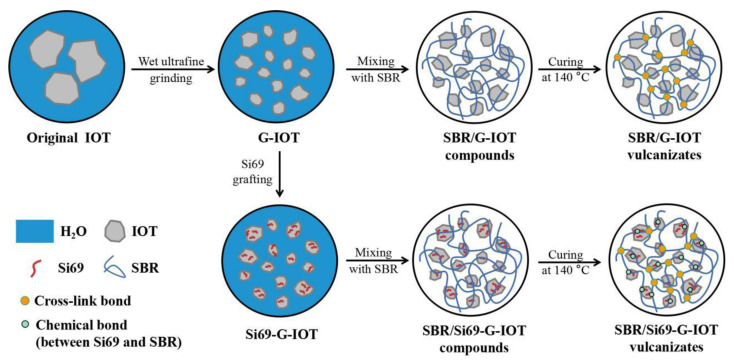
Schematic diagram of the SBR composite preparation process.

SBR composites were prepared according to the formulation shown in Table 2. SBR was masticated in a TY-160 two-roll mixing mill (Jiangdu Tianyuan Testing Machinery Co., Ltd., Yangzhou, China). Then, antioxidant 4010NA, G-IOTs/Si69-G-IOTs, zinc oxide, stearic acid, accelerator M, accelerator TMTD, aromatic hydrocarbon oil and sulfur were added in order to obtain SBR/G-IOT or SBR/Si69-G-IOT compounds, which were mixed well and placed in film for 24 h. The SBR/G-IOT or SBR/Si69-G-IOT vulcanizates were obtained by curing the SBR/G-IOT or SBR/Si69-G-IOT compounds in a vulcanizate mold (thickness of 2 mm) using a TY-2500 electrothermal press vulcanizer (Jiangdu Tianyuan Testing Machinery Co., Ltd., Yangzhou, China) under 14 MPa pressure at 140 °C for the optimum cure time. Figure 1 presents a schematic diagram of the SBR composite preparation process.

### 2.5. Model of Grinding Simulation

A population balance model (PBM) is a mathematical tool that can calculate the breakage behavior of particles with a given particle size. Austin [38] proposed the approximate formula of the basic integral differential equation of PBM in the grinding process as Equation (2).

(2) $$\frac{{\mathrm{dN}}_{i}\left(t\right)}{\mathrm{dt}}=-S_{i}N_{i}\left(t\right)+\overset{\infty }{\underset{j=1}{\sum }}S_{j}b_{i,j}N_{j}\left(t\right),\;i=1,\;2,\;3\ldots n$$

The size of the particle is divided into n levels, from coarse to fine, within a certain range, and i,j represents any one of 1 − n. t is the grinding time; N_i_(t) represents the mass fraction of particles with size i when the grinding time is t; S_i_ represents the breakage rate of the particles with size i under a certain stress; and b_i,j_ is the breakage function, which represents the percentage of the volume of particles that change to size i after a particle of size j is broken.

(3) $$\frac{{\mathrm{dR}}_{i}\left(t\right)}{\mathrm{dt}}=-S_{i}R_{i}\left(t\right)+\overset{\infty }{\underset{j=1}{\sum }}[(S_{j+1}B_{i,j+1}-S_{j}B_{i,j})R_{i}\left(t\right)],\;i=1,\;2,\;3\ldots n$$

Equation (3) is the cumulative form of Equation (1). $R_{i}\left(t\right)=\overset{i}{\underset{j=1}{\sum }}N_{j}\left(t\right)$ represents the cumulative oversized mass fractions of particle size i after t time grinding. $B_{i,j}=\overset{i}{\underset{j=1}{\sum }}b_{k,j}$ is the cumulative breakage distribution function, which represents the probability of fragments formed by breakage of particles in size class j to have a size less than i. The solution of Equation (3) is used to describe the particle size distribution of the grinding process.

(4) $$\ln\frac{R_{i}\left(t\right)}{R_{i}\left(0\right)}\approx K_{i}^{\left(1\right)}t$$

Equation (4) represents an approximate solution of Equation (3). $K_{i}^{\left(1\right)}$ is the first Kapur function, which is a slope value obtained by linear fitting of Equation (4) [39] and is the variable about Equations (5) and (6) [40,41].

(5) $$S_{i}=-K_{i}^{\left(1\right)}$$

(6) $$b_{i,j}=\frac{K_{i-1}^{\left(1\right)}-K_{i}^{\left(1\right)}}{K_{j}^{\left(1\right)}}$$

## 3. Results and Discussion

### 3.1. Particle Size Distribution and Specific Surface Area of G-IOTs

Generally, the specific surface area and particle size distribution of filler have a significant effect on rubber reinforcement. The rubber molecular chain forms a stronger link with particles of smaller size and larger specific surface area, which results in stronger adsorption capacity [42,43]. Narrower particle size distribution can reduce the influence of uneven-particle-size filler on the mechanical properties of rubber. Thus, we explored the effects of ball milling speed and grinding time on the specific surface area and particle size distribution of G-IOTs.

**Figure 2:**
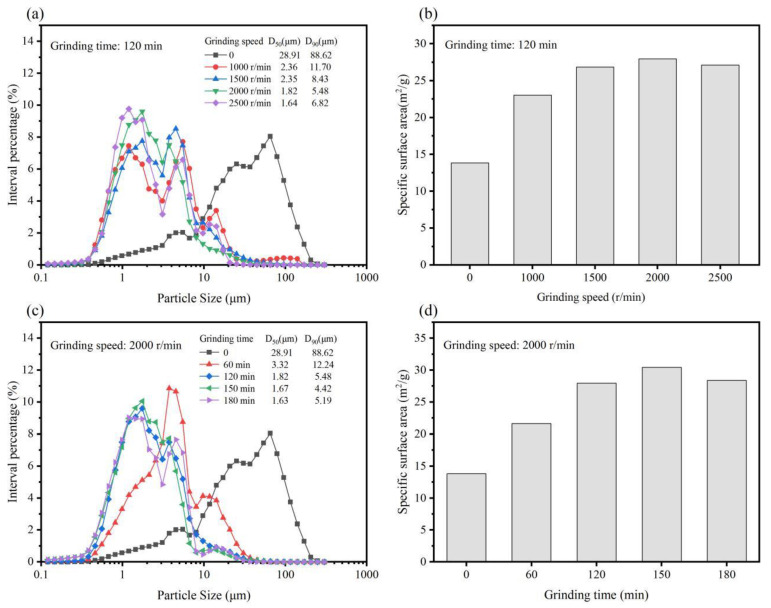
(**a**) Particle size distributions and (**b**) specific surface area of G-IOTs with different grinding speeds. (**c**) Particle size distributions and (**d**) specific surface area of G-IOTs with different grinding times.

Figure 2a,b shows the effect of ball mill grinding speed on the particle size distribution and specific surface area of G-IOTs when the grinding time was 120 min. The particle size distribution of G-IOTs narrowed, and the specific surface area increased, as the ball milling speed increased. The minimum value of D_90_ was 5.48 μm, and the maximum value of surface area was 28.31 m^2^/g, when the ball mill grinding is 2000 r/min. Figure 2c,d shows the effect of the ball milling speed on the particle size distribution and specific surface area of G-IOTs when the ball milling speed was 2000 r/min. Within 60 min of grinding time, the particle size of the G-IOTs was greatly reduced, and when the time continued to increase, the particle size distribution of the G-IOTs was further narrowed, and the specific surface area further increased. Grinding for 150 min, the minimum value of D_90_ was 4.42 μm, and the maximum value of surface area was 30.43 m^2^/g. Interestingly, the value of D_90_ increased, and the value of the specific surface area decreased, when the grinding speed was greater than 2000 r/min and the grinding time greater than 150 min. This was due to small G-IOT particles agglomerating with themselves or adsorbing onto large particles. Therefore, ball mill grinding at a speed of 2000 r/min for 150 min is the most suitable condition to produce rubber filler.

### 3.2. Simulation and Grinding Mechanism of IOTs

The production of filler in industry generally requires the grinding technology of powder, which consumes energy and causes production costs. Therefore, it is worth thoroughly studying the grinding mechanism of grinding IOTs with a ball mill, which could not only provide a theoretical basis for further refining and size classification of iron tailing particles but avoid excessive energy consumption in industrial production. In this paper, the particle size and grinding time of IOT were investigated with a first-order dynamic simulation using the PMB model under the conditions of grinding speeds of 1000 r/min and 2000 r/min.

**Figure 3:**
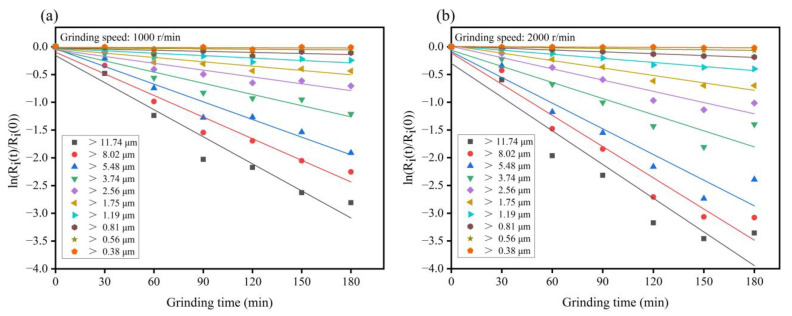
The linear fitting results of the ground residual fraction of G-IOT particle size and grinding time under grinding speeds of (**a**) 1000 r/min and (**b**) 2000 r/min.

As shown in the Table 3, the particle size of IOT was divided into 10 levels, from large to small, and the ratio of the upper and lower limits of each level was about 1.21. Figure 3a,b shows the linear fitting results of the ground residual fraction of G-IOT particle size and grinding time under the conditions of grinding speed of 1000 r/min and 2000 r/min, respectively. The slopes of the straight lines in Figure 3a,b are the Kapur function values under the corresponding particle sizes, and the Si value was calculated by Equation (5).

**Figure 4:**
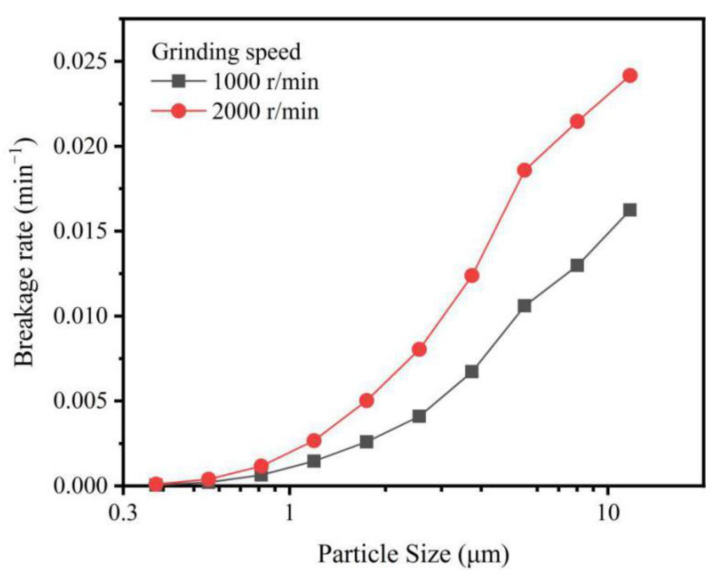
The breakage rates of IOTs with different particle sizes at grinding speeds of 1000 r/min and 2000 r/min.

Figure 4 shows the relationship between the breakage rate (S_i_) and particle size of IOTs, which can be used to evaluate the probability of selected breakage of particles with different particle sizes. The S_i_ value increased significantly as the particle size of IOT increased, indicating that IOTs with larger particle size were easier to break. Furthermore, IOTs at the grinding speed of 2000 r/min had a higher S_i_ value than those at the grinding speed of 1000 r/min, especially with larger particle sizes. This result indicates that increasing the grinding speed increased the breakage probability of larger IOT particles, and it was almost ineffective when the particle sizes of IOTs were smaller than 1 μm. Two reasons may be responsible. First would be the stronger impact on IOTs with increasing grinding speed, which would be attributable to the greater contact area between the grinding balls and IOTs in which the particle size is large. Meanwhile, increasing grinding speed would have little effect if the particle size of the IOTs was too small and the grinding balls applied only shear force to them [44]. Second would be the higher surface free energy of G-IOTs, due to the broken bonds and unsaturated bonds produced during the grinding process, making small G-IOT particles agglomerate with themselves or adsorb onto large particles [45]. To further reduce the particle size of IOTs, increasing the grinding speed while reducing the size of grinding balls or adding dispersant is a possibility.

In the grinding process, the properties and strength of the stress on the particles affect the breakage process. There are three main grinding mechanisms of particles [42,46]: (1) abrasion—in this case, the particles are generally subjected to stress in the shear direction, the broken particles produce a bimodal particle size distribution, and the particle size after grinding is close to the initial particle size; (2) cleavage—in this case, a strong stress is applied slowly to the particles, resulting in fragments 50–80 vol% smaller than the initial particle size; and (3) fracture—this is generally caused by the rapid application of stress to the particles, and the particle size of the fragments is 20–70 vol% of the initial particle size.

(7) $$b_{i.j}=\left(\begin{array}{cccccccccc} 0 & 0 & 0 & 0 & 0 & 0 & 0 & 0 & 0 & 0\\ 0.1117 & 0 & 0 & 0 & 0 & 0 & 0 & 0 & 0 & 0\\ 0.1192 & 0.1341 & 0 & 0 & 0 & 0 & 0 & 0 & 0 & 0\\ 0.2565 & 0.2888 & 0.3335 & 0 & 0 & 0 & 0 & 0 & 0 & 0\\ 0.1796 & 0.2021 & 0.2335 & 0.3503 & 0 & 0 & 0 & 0 & 0 & 0\\ 0.1249 & 0.1407 & 0.1625 & 0.2437 & 0.3752 & 0 & 0 & 0 & 0 & 0\\ 0.0976 & 0.1099 & 0.1269 & 0.1905 & 0.2932 & 0.4692 & 0 & 0 & 0 & 0\\ 0.0620 & 0.0699 & 0.0806 & 0.1211 & 0.1863 & 0.2982 & 0.5618 & 0 & 0 & 0\\ 0.0319 & 0.0359 & 0.0415 & 0.0622 & 0.0958 & 0.1533 & 0.2887 & 0.6589 & 0 & 0\\ 0.0119 & 0.0134 & 0.0154 & 0.0231 & 0.0356 & 0.0570 & 0.1074 & 0.2450 & 0.7185 & 1 \end{array}\right)$$

b_i,j_ represents the mass fraction of particles that change to size i after the particles of size j are broken. Equation (7) is the breakage function matrix calculated under the condition of IOT grinding speed of 2000 r/min. b_i,j_ had larger values when j = 1−2 and i < 5. This means that most G-IOT particle sizes changed to 3.47–5.48 μm when the IOT particle size was beyond 8.02 μm, indicating that the main grinding mechanisms were cleavage and fracture. The value of b_i,j_ increases gradually as j increased when j = 3−6 and i < j + 2, indicating that when the particle sizes of IOTs were 1.75–8.02 μm, the smaller the IOT particle size was, the closer the size of the G-IOT particles was to that of the IOT particles, and the further the grinding mechanism gradually transitioned from cleavage and fracture to abrasion. The value of b_i,j_ was greater than 0.55 when j = 7−10 and i = j + 1. This means that most G-IOT particle sizes were close to IOT particle sizes, i.e., 0.38–1.75 μm, and that the grinding mechanism was abrasion. According to Figure 2c, the main grinding mechanisms were cleavage and fracture when grinding time was less than 60 min, and these gradually transitioned to abrasion as grinding time increased. This result also explains the significant bimodal particle size distribution of G-IOTs in Figure 2c when the grinding time was beyond 120 min.

### 3.3. X-ray Diffraction Analysis of G-IOTs

**Figure 5:**
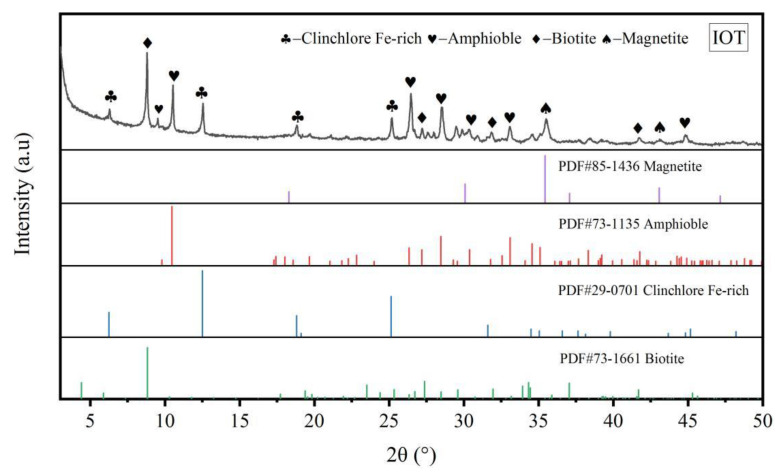
X-ray diffraction pattern of IOTs.

Figure 5 shows the X-ray diffraction pattern of IOTs. The main mineral composition of the IOTs was made up by silicate minerals, which mainly included amphibole, clinochlore (Fe-rich), biotite and magnetite.

**Figure 6:**
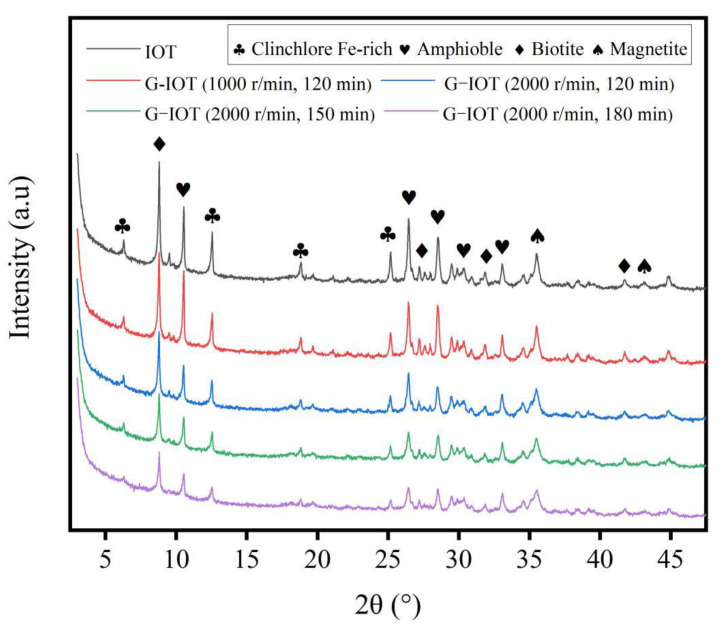
X-ray diffraction patterns of G-IOTs with different grinding speeds and grinding times.

It can be seen in Figure 6 that ball mill grinding speed and grinding time did not change the mineral composition of G-IOTs. The diffraction peak of each mineral crystal in the G-IOTs became weaker compared with the corresponding peak in the IOTs. This weakening increased with grinding speed and grinding time, and the diffraction peaks of clinochlore (Fe-rich) at 2θ = 6.229° and 2θ = 18.765° gradually disappeared. This indicates that the grinding process transformed part of the IOT mineral crystals into an amorphous form, and that grinding refined the crystal grains and decreased the crystallinity of the G-IOTs [47,48]. The half-peak width of the G-IOTs increased with grinding speed and grinding time. This means that the strain in the lattice of the G-IOTs increased continuously, indicating that the G-IOT grains were more active because of the occurrence of stress defects or lattice defects in the grain caused by grinding [49]. All the changes to G-IOT crystals occurring during the grinding process made it store more energy to form activation points, which provided the chemical environment for the organic modification of G-IOTs in the next step.

### 3.4. Fourier Transform Infrared Spectroscopy of Si69-G-IOTs

**Figure 7:**
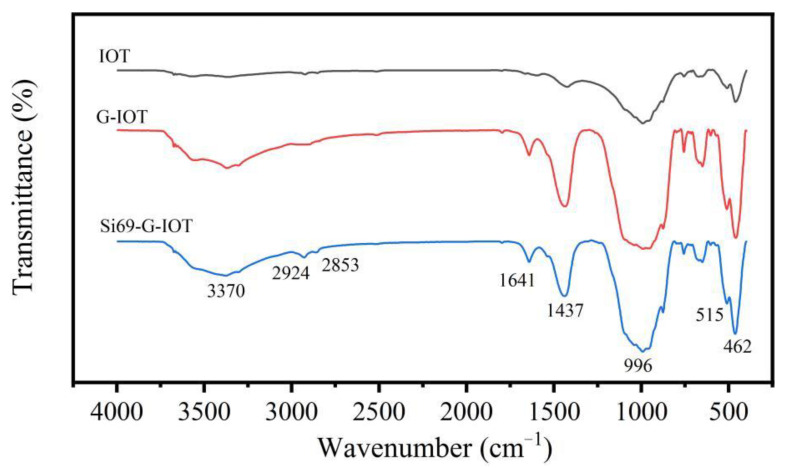
FTIR spectra of IOTs, G-IOTs and Si69-G-IOTs.

Figure 7 shows the FTIR spectra of IOTs, G-IOTs and Si69-G-IOTs. The G-IOTs generated new stretching vibration absorption peaks of OH (H_2_O) at 3370 cm^−1^ and 1641 cm^−1^ compared with the IOTs, indicating that crystal water and adsorbed water were produced on the G-IOTs’ surface during the wet ultrafine grinding process. The stretching vibration absorption peaks of IOT and G-IOT at 996 cm^−1^, 515 cm^−1^ and 462 cm^−1^ were formed by Si-O and Si-O-Si bonds, which became stronger after grinding. This result was due to the long-range ordered structure of IOT crystals being destroyed by grinding, which further increased the number of broken Si-O bonds in the G-IOTs. All the changes to the stretching vibration absorption peaks of the G-IOTs provided the chemical environment for organic modification. The new stretching vibration absorption peaks of CH_2_ and CH_3_ appeared at 2924 cm^−1^ and 2853 cm^−1^ in the Si69-G-IOTs, and the peaks of the Si-O bonds at 996 cm^−1^ and 462 cm^−1^ and the peaks of OH (H_2_O) at 3370 cm^−1^ and 1641 cm^−1^ were slightly weaker in the Si69-G-IOTs than in the G-IOTs. This indicates that part of the OH group in the G-IOTs reacted with Si69 to form chemical bonds, further confirming that Si69 was successfully grafted to the surface of the G-IOTs.

### 3.5. Cure Characteristics of SBR Composites

**Figure 8:**
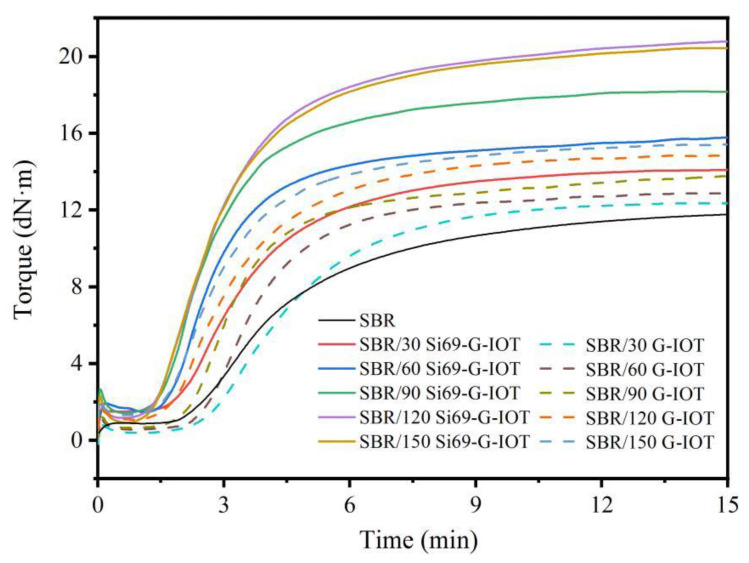
Cure curves of SBR composites.

The cure curves of SBR composites are shown in Figure 8. Increase in filler loading levels and Si69 modification of G-IOTs led to increases in the maximum torque of SBR composites. This may be related to the limited slip motion of the SBR molecular chain.

The maximum and minimum torque difference (M_H_−M_L_) is direct proportion to the cross-link density of rubber. Table 4 shows that the M_H_-M_L_ and CRI values of SBR/Si69-G-IOT were significantly greater than those of SBR/G-IOT under the same filler loading level, because the sulfur group in Si69 that was grafted on the G-IOTs could participate in vulcanization process of SBR/Si69-G-IOT to accelerate the vulcanization reaction [50]. The M_H_-M_L_ value of SBR/Si69-G-IOT increased as the filler loading increased, meaning that the cross-link density of SBR/Si69-G-IOT increased. Interestingly, the CRI value of SBR composites increased as the filler loading increased. This result may be due to the significant thermal conductivity of IOT assisting in the vulcanization reaction of SBR composites [32].

### 3.6. Static Mechanical Properties of SBR Composites

**Figure 9:**
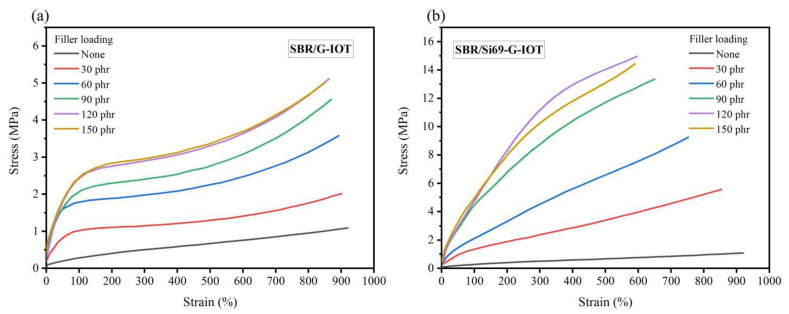
Stress−strain curves of (**a**) SBR/G-IOT and (**b**) SBR/Si69-G-IOT.

Figure 9 shows the stress–strain behaviors of SBR composites with different filler loading levels. As Figure 9a shows, the stress of SBR/G-IOT increased rapidly in the strain range of 0 to 150%. After that, the SBR composites incurred stress yield. This may have been due to the weaker interaction between the G-IOTs and the SBR molecular chain leading to the rubber molecular chain on the surface of the G-IOTs slipping under the external force. It can be observed from Figure 9b that the stress–strain behavior of SBR/Si69-G-IOT was greatly improved. This may have been due to the stronger interaction between Si69 and the SBR molecular chain preventing the slipping of rubber molecules.

**Figure 10:**
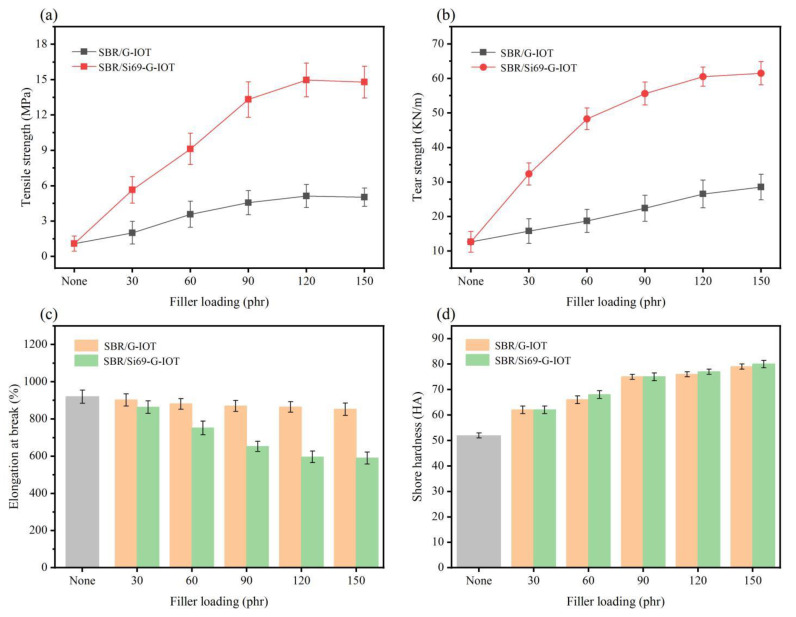
(**a**) Tensile strength, (**b**) elongation at break, (**c**) tear strength and (**d**) shore hardness of SBR composites.

Figure 10 shows the effect of G-IOT/Si69-G-IOT loading level on the mechanical properties of SBR composites. As Figure 10a,b shows, the tensile strength and tear strength of the SBR composites greatly improved after adding Si69. This may have been for two reasons: (1) Si69 increasing the surface activity of the G-IOTs, which would reduce the difference in surface energy between the filler and rubber so that the filler could be better dispersed in the rubber; (2) in the vulcanization process, the polysulfide bond of Si69 in the Si69-G-IOTs breaking to form sulfur-containing free radicals that would graft on the SBR molecular chain to increase the interaction between filler and rubber [51]. The tensile strength and tear strength of the SBR composites increased continuously as the filler loading increased. It increased rapidly when the filler loading was 30–90 phr, and the increase slowed down when the filler loading was greater than 90 phr. This may have been due to excessive filling, which would have led to an increase in the force between filler and filler in the composite rubber. The tensile strength reached a maximum value in SBR/120 Si69-G-IOT of 14.97 MPa, and the tear strength of SBR/150 Si69-G-IOT was 61.49 KN/m. 

As shown in Figure 10c, the elongation at break of SBR/Si69-G-IOT was significantly lower than that of SBR/G-IOT under the same filler loading level, which decreased and stabilized with increasing Si69-G-IOT loading. This result was attributed to the increase in the cross-linking density of SBR/Si69-G-IOT, which limited the sliding of the rubber macromolecular chain. It can be seen in Figure 10d that there was not much difference in shore hardness between SBR/Si69-G-IOT and SBR/G-IOT under the same filler loading, and that shore hardness rose as filler loading increased. This shows that the hardness of SBR composites mainly depended on the filler loading, and the addition of Si69 had no effect.

### 3.7. Morphology Characteristics of SBR Composites

**Figure 11:**
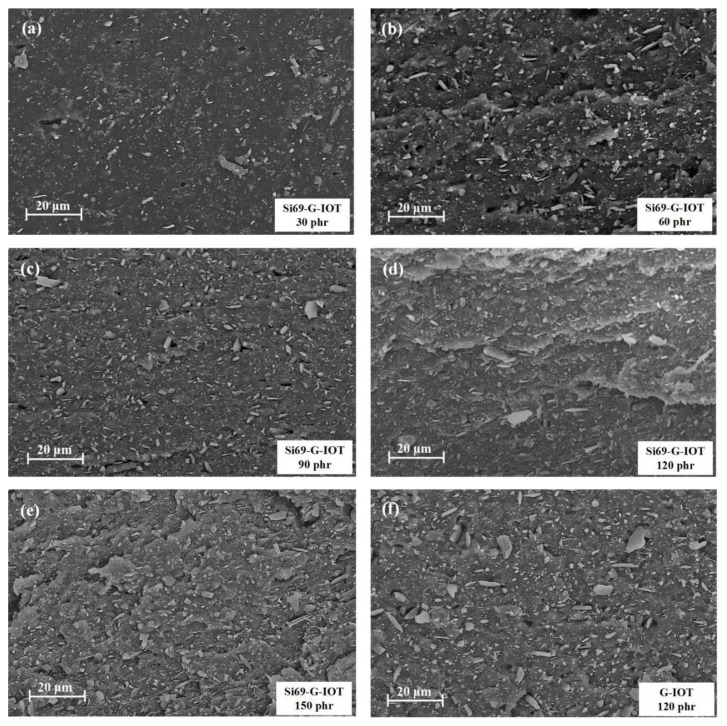
SEM image of the tensile sections of (**a**) SBR/30 Si69-G-IOT, (**b**) SBR/60 Si69-G-IOT, (**c**) SBR/90 Si69-G-IOT, (**d**) SBR/120 Si69-G-IOT, (**e**) SBR/150 Si69-G-IOT, (**f**) SBR/120 G-IOT.

The tensile strength of rubber is closely related to the distribution of filler particles in the rubber matrix. The morphology of the tensile sections of the SBR composites filled with Si69-G-IOT loading from 30 to 150 phr are shown in Figure 11a–e. The Si69-G-IOT was evenly distributed in the rubber matrix, and the crack in the layer of the SBR composite section became more obvious as the Si69-G-IOT loading increased. This effect indicates that the interaction between the SBR matrix and the Si69-G-IOT increased. As shown in Figure 11d,f, compared with that of SBR/120 Si69-G-IOT, the tensile section of SBR filled with 120 phr G-IOTs had no obvious cracks, while more filler was pulled out. This result proved that Si69 significantly increased the bonding between the filler and the SBR matrix.

**Figure 12:**
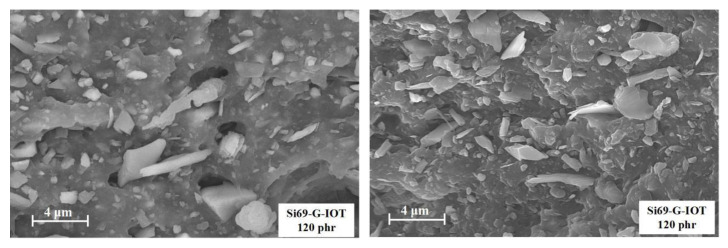
SEM image of SBR/120 Si69-G-IOT.

Since SBR/120 Si69-G-IOT had the maximum value of tensile strength. It was selected for further study in the form of Si69-G-IOT distribution in the fracture surface of rubber. Figure 12 shows that the Si69-G-IOTs were irregularly shaped sheets and arranged perpendicular to the fracture surface. The small-size Si69-G-IOTs were tightly combined with the SBR matrix, and a small number of large-size Si69-G-IOTs were pulled out during the process of breaking because of poor adhesion with rubber, resulting in holes in the SBR matrix.

### 3.8. Dynamical Mechanical Analysis of SBR Composites

**Figure 13:**
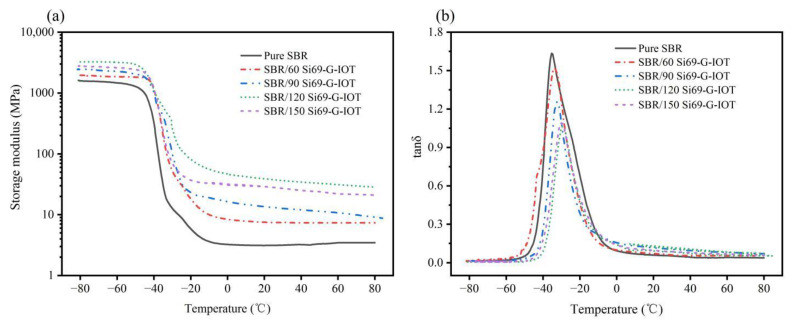
The temperature dependence of (**a**) storage moduli and (**b**) loss factors (tanδ) of SBR composites with different filler loading.

Dynamic mechanical analysis can be used to study the interaction between the filler and the rubber matrix. Figure 13a shows the temperature dependence of the storage modulus of SBR composites in a range of −80 to 80 °C. Storage modulus reflects the ability of rubber material to restore elasticity under stress. The storage modulus the of SBR composites decreased sharply around −45 °C and decreased gradually around −20 °C. This was due to the SBR/Si69-G-IOT main chain’s transition from the glassy state to the rubbery state. The values of the storage moduli of SBR composites at different temperature are shown in Table 5. The storage modulus of the SBR composites increased as the filler loading increased from 60 to 120 phr at all temperatures. For example, in the low-temperature region (−60 °C), the SBR storage modulus was 1469.05 MPa, while the SBR/120 Si69-G-IOT storage modulus increased to 3186.83 MPa. This shows that the higher filler loading increased the ability to restore elasticity of SBR composites, which may have been due to the increase in the composites’ cross-link density and stiffness.

Figure 13b shows the temperature dependence of the loss factor (tanδ) of SBR composites in a range of −80 to 80 °C. The tanδ of SBR composites was close to 0 and then showed a sharp peak as temperature increased. This effect was due to the molecular chain of SBR composites freezing at low temperature. The chain began to move as the temperature increased, and this caused the loss of energy. The decrease in the tanδ peak was due to the stronger interface between the filler and rubber. It can be seen from Figure 13b that the tanδ peak heights of SBR/Si69-G-IOT were reduced compared with those of SBR. As shown in Table 5, the tanδ value of SBR composites decreased as the Si69-G-IOT loading increased, and SBR/120 Si69-G-IOT had the smallest tanδ value at 1.05, while the tanδ value of SBR was 1.63. This result shows that increase in Si69-G-IOT loading made a stronger interface combination between Si69-G-IOT and the SBR matrix. The temperature corresponding to the maximum value of tanδ is the glass transition temperature (T_g_) of rubber. As shown in Table 5, the T_g_ of SBR composites all moved to higher temperatures. The T_g_ of SBR/120 Si69-G-IOT was the maximum, at −29.29 °C, and the T_g_ of SBR was −35.38 °C. This shows that adding more Si69-G-IOT could better inhibit the movement of the SBR molecular chain and reduce the internal energy loss caused by the friction of the rubber molecular chain.

## 4. Conclusions

In this paper, significant mechanical properties of rubber composites were achieved by filling an SBR matrix with Si69-G-IOT prepared using wet ultrafine grinding and surface organic modification techniques. SBR/120 Si69-G-IOT showed excellent tensile strength, with a value of 14.97 MPa, while maintaining high elongation at break, with a value of 596.36%. G-IOTs reached the narrowest particle size distribution at a grinding speed of 2000 r/min for 150 min, with a D_90_ value of 4.419 μm and a specific surface area value of 30.43 m^2^/g. By reacting with OH bonds on G-IOTs produced by grinding, Si69 was successfully grafted on G-IOTs, significantly enhancing the cure characteristics and mechanical properties of SBR/Si69-G-IOT compared with those of SBR/G-IOT. Static mechanical and dynamic mechanical properties of SBR composites increased as the filler loading increased because of the increase in interaction between the filler and the SBR matrix. These results show that it is feasible to use a large amount of accumulated pollutant IOTs to prepare rubber-reinforcing filler, which could probably also be applied to the reinforcement of other kinds of rubber. This work not only reduced the production cost of rubber but provided a new method of resource utilization for iron tailings. It is worth noting that since iron tailings contain certain amounts of magnetite and hematite, the rubber composites have the prospect of realizing magnetic functionalization, which could be used in automobiles and electronic equipment. However, we found that the poor contact between large-size G-IOT particles (>4 μm) and rubber matrix might be the key factor limiting the mechanical properties. It is necessary to improve the ultrafine grinding process and optimize the size classification technology.

## Figures and Tables

**Table 1:** The chemical components of the IOTs.

Compound	SiO_2_	Fe_2_O_3_	CaO	MgO	Al_2_O_3_	K_2_O	Na_2_O	TiO_2_
Concentration (wt%)	39.0	19.1	14.2	11.2	9.87	1.53	1.01	1.65

**Table 2:** The compound formula of SBR composites.

Samples	Ingredients (phr)									
	SBR	G-IOT	Si69-G-IOT	4010NA	Zinc Oxide	Stearic Acid	M	TMTD	Sulfur	Aromatic Hydrocarbon Oil
Pure SBR	100	0	0	2	3	1	0.6	0.6	1.8	3
SBR/30 G-IOT	100	30	0	2	3	1	0.6	0.6	1.8	3
SBR/60 G-IOT	100	60	0	2	3	1	0.6	0.6	1.8	3
SBR/90 G-IOT	100	90	0	2	3	1	0.6	0.6	1.8	3
SBR/120 G-IOT	100	120	0	2	3	1	0.6	0.6	1.8	3
SBR/150 G-IOT	100	150	0	2	3	1	0.6	0.6	1.8	3
SBR/30 Si69-G-IOT	100	0	30	2	3	1	0.6	0.6	1.8	3
SBR/60 Si69-G-IOT	100	0	60	2	3	1	0.6	0.6	1.8	3
SBR/90 Si69-G-IOT	100	0	90	2	3	1	0.6	0.6	1.8	3
SBR/120 Si69-G-IOT	100	0	120	2	3	1	0.6	0.6	1.8	3
SBR/150 Si69-G-IOT	100	0	150	2	3	1	0.6	0.6	1.8	3

**Table 3:** Size classes of IOT particles.

Class i,j	1	2	3	4	5	6	7	8	9	10
Size range/μm	>11.74	11.74–8.02	8.02–5.48	5.48–3.74	3.74–2.56	2.56–1.75	1.75–1.19	1.19–0.81	0.81–0.56	0.56–0.38

**Table 4:** Cure characteristics of SBR composites with different filler loading.

Filler Loading		t_02_ (min)	t_90_ (min)	M_H_−M_L_ (dN·m)	CRI (min^−1^)
None		2.17	10.10	11.67	12.61
30 phr	G-IOT	2.57	8.30	11.92	17.45
	Si69-G-IOT[M1]	1.73	6.25	12.90	22.12
60 phr	G-IOT	2.15	6.27	12.05	24.27
	Si69-G-IOT	11.50	5.10	14.61	27.78
90 phr	G-IOT	1.85	5.82	12.14	25.19
	Si69-G-IOT	1.32	4.45	16.46	31.95
120 phr	G-IOT	1.51	5.27	13.52	26.60
	Si69-G-IOT	1.35	4.10	19.67	36.36
150 phr	G-IOT	1.42	4.93	13.57	28.49
	Si69-G-IOT	1.30	3.73	19.83	41.15

**Table 5:** Analysis of the dynamic mechanical properties of SBR composites.

Samples	Storage Modulus (MPa) at Different Temperatures					T_g_ (°C)	tanδ
	−60 (°C)	−30 (°C)	0 (°C)	30 (°C)	60 (°C)		
SBR	1469.05	12.32	3.24	3.15	3.46	−35.38	1.63
SBR/60 Si69-G-IOT	1851.21	52.63	8.40	7.46	7.34	−33.28	1.52
SBR/90 Si69-G-IOT	2262.04	100.37	16.45	12.67	10.72	−32.25	1.25
SBR/120 Si69-G-IOT	3186.83	257.15	46.66	36.30	31.14	−29.29	1.05
SBR/150 Si69-G-IOT	2591.94	69.94	30.68	27.32	22.63	−29.93	1.08

## Data Availability

All the data is available within the manuscript.
